# Ontogenesis of Aldehyde Pheromones in Two Synanthropic Bed Bug Species (Heteroptera: Cimicidae)

**DOI:** 10.3390/insects11110759

**Published:** 2020-11-05

**Authors:** Mark Dery, Kyle Arriola, Chow-Yang Lee, Dong-Hwan Choe

**Affiliations:** 1 Department of Entomology, University of California, Riverside, CA 92521, USA; chowyang.lee@ucr.edu (C.-Y.L.); donghwan.choe@ucr.edu (D.-H.C.); 2 Department of Chemistry, University of California, Riverside, CA 92521, USA; karri003@ucr.edu

**Keywords:** bed bug, *Cimex lectularius*, *Cimex hemipterus*, aldehyde, ontogenesis

## Abstract

**Simple Summary:**

The common (*Cimex lectularius*) and tropical (*C. hemipterus*) bed bugs are widespread blood-feeding human pests. To better understand their chemical ecology, the current study quantified the four most abundant bed bug aldehydes in the bed bugs’ immature stages by extracting their freshly shed exuviae. We also quantified the two most abundant aldehydes in adult bed bugs via surface extraction. Quantitative comparisons are made across different developmental stages (nymph), species (nymph and adult), or sexes (adult). In immature bed bugs, significant quantitative differences of four aldehydes were found across developmental stages within a species, and between species within a stage. Similarly, in adult bed bugs, a significant difference in the ratio of (*E*)-2-hexenal and (*E*)-2-octenal was found between species. This study provides the first systematic quantification of these aldehydes for all five of the nymphal stages for both *C. lectularius* and *C. hemipterus*.

**Abstract:**

Bed bugs produce volatile aldehydes that have alarm and aggregation functions. Using two synanthropic bed bug species, *Cimex lectularius* L. and *C. hemipterus* (Fabricius), developmental changes were examined for (*E*)-2-hexenal, 4-oxo-(*E*)-2-hexenal, (*E*)-2-octenal, and 4-oxo-(*E*)-2-octenal, the four most abundant aldehydes shared between the two species. Quantitative analyses of the aldehydes in the nymphal exuviae indicated that the aldehydes’ ratio remained similar throughout nymphal development. In general, (*E*)-2-octenal was most abundant, and (*E*)-2-hexenal and 4-oxo-(*E*)-2-octenal were least abundant. The fourth aldehyde, 4-oxo-(*E*)-2-hexenal, was present in intermediate quantities. The quantities and percent abundances of the aldehydes in nymphal exuviae and the adults were significantly different between *C. lectularius* and *C. hemipterus*. The ratio between (*E*)-2-hexenal and (*E*)-2-octenal was determined in adult male and female bed bugs of each species. Adult *C. hemipterus* had a higher proportion of (*E*)-2-hexenal than *C. lectularius*, while no sex differences were found. This work provides the first systematic quantification of four aldehydes [(*E*)-2-hexenal, 4-oxo-(*E*)-2-hexenal, (*E*)-2-octenal and 4-oxo-(*E*)-2-octenal] for all five of the nymphal stages for both *C. lectularius* and *C. hemipterus*.

## 1. Introduction

Bed bugs (Hemiptera: Cimicidae) are ectoparasites that are obligate blood-feeders [1]. The common bed bug, *Cimex lectularius* L., and the tropical bed bug, *Cimex hemipterus* (Fabricius), are considered urban pests, predominantly utilizing humans as hosts. *Cimex lectularius* has a worldwide distribution, while *C. hemipterus* inhabits both tropical and subtropical regions [1]. *Cimex lectularius* and *C. hemipterus* diverged from each other ≈ 47 MYA and are descended from bat and bird associated lineages, respectively [2]. *Cimex hemipterus* have been recently reported in Florida for the first time since the bed bug resurgence [3] and for the first time in Hawaii [4]. These reports suggest the possibility of *C. hemipterus* further expanding outside of their historical range. While the range of *C. lectularius* and *C. hemipterus* overlap [5,6] and the two species can mate with each other, viable hybrids are not typically produced [1,7].

The chemical ecology of bed bugs, particularly *C. lectularius*, has been the subject of many investigations due to its potential use for monitoring and control [8]. *Cimex lectularius* is known to respond to conspecific cuticular hydrocarbons, exuviae, used harborage paper, feces, and associated volatiles [9,10,11,12]. A distinctive odor associated with *C. lectularius* is caused by several aldehydes that they produce [13]. *Cimex lectularius* nymphs produce the aldehydes in their dorsal abdominal glands while the adults produce the aldehydes in their metathoracic scent glands [1,14,15,16]. Two of these aldehydes, (*E*)-2-hexenal and (*E*)-2-octenal, are released in relatively large quantities when bed bugs are attacked by predators (e.g., bats or ants) or conspecifics (e.g., mating attempts by adult males), serving an alarm/defensive purpose [1,17,18,19]. (*E*)-2-hexenal and (*E*)-2-octenal are two of the compounds that comprise the *C. lectularius* aggregation pheromone [20]. When these aldehydes are present at lower concentrations relative to the amount released during an alarm response, they appear to function as an aggregation pheromone, encouraging bed bugs to form large groups in their harborage sites [9,11,20,21]. 

In addition to (*E*)-2-hexenal and (*E*)-2-octenal, two ketoaldehydes [4-oxo-(*E*)-2-hexenal and 4-oxo-(*E*)-2-octenal] have been found in the dorsal abdominal glands of nymphs [14]. These ketoaldehydes are not known to occur in adult bed bugs. The ketoaldehydes made up at least 16% of the total aldehydes detected in the dorsal abdominal gland contents of the fourth and fifth instars of *C. lectularius* [14]. The dorsal abdominal gland reservoirs are shed during molting, and the aldehydes slowly volatilize from the gland reservoirs in the exuviae [9]. As developing bed bugs would continue to produce a large amount of exuviae, the accumulated exuviae in the harborage site may contribute to the formation of aggregations [9]. 

Compared to *C. lectularius*, less is known on the chemical ecology of *C. hemipterus*. A headspace collection study with *C. hemipterus* reported the presence of the four aldehydes mentioned above [22]. As with *C. lectularius*, (*E*)-2-hexenal, and (*E*)-2-octenal were emitted by both adults and late instar nymphs. In contrast, the ketoaldehydes were emitted only by nymphs [22]. Liedtke et al. [22] also found that whole-body extract of late instar *C. hemipterus* nymphs was repellent to conspecific adults and nymphs. In addition, among 33 compounds identified from the excreta of *C. hemipterus*, (*E*)-2-hexenoic acid, hexanal, and (*E*)-2-hexenal elicited aggregation responses in both adults and nymphs [23].

As most of the existing literature on bed bug aldehydes focused on late instar nymphs or adults, information on the aldehyde production in earlier nymphal stages and their quantitative change across development are currently lacking for these two species. To further our knowledge on the production of the bed bug aldehydes, the current study investigates these aldehydes’ ontogenesis in two synanthropic bed bugs, *C. lectularius*, and *C. hemipterus*. For both species, nymphal exuviae were used to quantify the aldehydes in the first, second, third, fourth, and fifth instar nymphs. Additionally, (*E*)-2-hexenal and (*E*)-2-octenal were quantified for both species’ female and male adults. 

## 2. Materials and Methods

### 2.1. Insects

Two species of bed bugs were used throughout the experiments. *Cimex lectularius* originated from colonies started from “Earl” strain individuals obtained from Sierra Research Laboratories (Modesto, CA, USA). The Earl strain was originally collected in Modesto, CA, in 2007. *Cimex hemipterus* were collected between 2005 and 2006 from multiple locations in Kuala Lumpur, Malaysia, imported into the US (CDC PHS Permit No. 03282018-11057, and 20190426-2698A), and reared in the quarantine facility of the University of California, Riverside. Bed bugs were kept in screened vials (9 cm in height, 4.5 cm in diameter) with a corrugated filter paper cylinder as a substrate. Bed bugs were fed using a custom glass feeder (Prism Research Glass, Inc., Raleigh, NC, USA), which allowed defibrinated rabbit blood (Hemostat Laboratories, Dixon, CA, USA) to be heated to 37 °C using a circulating water bath. Bed bugs were fed through a grafting tape membrane (Aglis & Co., Ltd., Yame City, Fukuoka, Japan) screen approximately every fourteen days. Colonies of *C. lectularius* were maintained at 24–26 °C and 15–30% RH, with a photoperiod of 12:12 (L:D) hours. Colonies of *C. hemipterus* were maintained at 22–23 °C and 40–60% RH, with a photoperiod of 12:12 (L:D) hours.

### 2.2. Nymphs (Solvent Extraction of Exuviae)

Aldehydes were quantified in *C. lectularius* and *C. hemipterus* nymphs by extracting from exuviae containing the reservoirs of shed dorsal abdominal glands. Exuviae were used as a substitute for whole nymphs due to the simplicity of extraction and sample clean-up compared with whole insects. As nymphs require a blood meal to molt [1], the timing of molting was controlled by feeding. For *C. lectularius*, unfed mixed instars were placed into a new colony vial and fed to repletion. After two days, the fed nymphs were placed individually into the wells (16 mm × 19 mm) of 24-well cell culture dishes (Corning Inc., Corning, NY, USA) lined with filter paper. The nymphal instar of *C. lectularius* was identified using a key to immature stages [1]. Since the nymphal instar key was not available for *C. hemipterus*, nymphs of *C. hemipterus* were raised from first instars to maturity, and the exuviae of each stage were collected. Exuviae were handled by the legs with fine-tipped metal forceps to ensure the shed dorsal abdominal gland reservoirs were not ruptured. Carbon dioxide was not used to anesthetize bed bugs to prevent the release of aldehydes. The bed bugs were observed at least twice daily for the presence of freshly shed exuviae, which were then collected and extracted within 24 h. 

The aldehydes in the exuviae were extracted using the following method adapted from Choe et al. [9]. First, exuviae were crushed in a glass tissue grinder containing 0.5 mL dichloromethane (DCM). Sulcatone (6-methyl-5-hepten-2-one; Sigma-Aldrich, St. Louis, MO, USA) was used as an internal standard (200 ng/mL). Due to small quantities of the compounds, pooled samples of five or two exuviae were used per sample for first and second instars, respectively. For other instars, a single exuvia was used per sample. Particulates were removed by filtering the extract through a glass pipette containing a glass wool plug. The filtered extract was collected into a 2 mL vial (Agilent Technologies, Santa Clara, CA, USA) and analyzed on the day of extraction. For both species, a total of 10 samples were obtained for each instar. Using an automatic liquid sampler (ALS) device, 1 µL of the extract was injected onto an Agilent 7890 gas chromatograph equipped with a DB-5 column (30 m × 0.25 mm inner diameter) and a flame ionization detector (GC-FID). Helium was used as the carrier gas. Samples were injected in splitless mode, with a temperature program of 50 °C for 1 min and then 10 °C min^−1^ to 280 °C with a 10-min hold. The compounds were identified based on a comparison of retention times with authentic standards. Standards of (*E*)-2-hexenal and (*E*)-2-octenal were purchased from Sigma-Aldrich, while 4-oxo-(*E*)-2-hexenal and 4-oxo-(*E*)-2-octenal were synthesized using the method described by Moreira and Millar [24]. 

### 2.3. Adults

For adult bed bugs, a surface extraction method was used to determine the percent abundance of (*E*)-2-hexenal and (*E*)-2-octenal. The surface extraction method and the use of relative quantity data were advantageous due to (1) the potential loss of the aldehydes during the sample clean-up process for crushed whole body extracts, and (2) the large amount of individual variation in the amount of aldehydes released/collected. Adult bed bugs were collected from a colony vial and placed individually into the wells of 24-well cell culture dishes lined with filter paper. Carbon dioxide was not used during the handling of bed bugs. To reduce aldehyde contamination by other colony members, the adults were kept isolated from the colony for 24 h before extraction. A single adult bed bug was placed into a 2-mL glass vial and anesthetized with carbon dioxide, causing the aldehydes to be released from the metathoracic scent glands onto the evaporating areas of the bed bug [1]. These released aldehydes were immediately collected by adding 0.5 mL DCM (containing 200 ng/mL sulcatone) into the vial containing the adult bed bug. After gentle swirling for approximately 5 s, 40 µL of the extract was removed and transferred into a new vial containing a glass insert (250 μL, Agilent Technologies, Santa Clara, CA, USA). The samples were analyzed using a GC-FID, as previously described for exuviae. The percent abundance of each compound was calculated based on the quantity of (*E*)-2-hexenal and (*E*)-2-octenal recovered. For each species, a total of 20 adults (10 male:10 female) were sampled. 

### 2.4. Quantification

The quantification of the aldehydes was effected with a calibration curve for each aldehyde (0.059, 0.118, 0.235, 0.469, 0.938, 1.875, 3.75, 7.5, and 15 µg/mL) versus sulcatone (200 ng/mL) as an internal standard, analyzed by GC-FID as described for exuviae. The ketoaldehydes’ calibration curves did not include the 0.059 µg/mL standard due to limited detection. For first and second instar exuviae, the resulting quantitative data were divided by the number of exuviae used for each sample (5 and 2 for first and second instar, respectively) to obtain the quantity data per exuvia. The percent abundance of each aldehyde was calculated using the total quantity of the four aldehydes. 

### 2.5. Statistical Analysis

Due to heteroscedasticity and non-normality in the quantitative data, nonparametric tests were used for the statistical analysis of aldehydes quantities. Comparisons across different instars within each species were carried out with Kruskal–Wallis H test followed by Dunn’s Multiple Comparisons with correction [25,26] using the R package Fisheries Stock Analysis [27]. For each instar, Wilcoxon rank-sum tests were used to compare the amounts of each aldehyde between species. Wilcoxon rank-sum tests were used to determine if species or sex significantly affected adult aldehyde percent abundance. For compounds that were not detected in a sample, a zero was used as quantity for the analyses.

A principal component analysis (PCA) was used to visualize differences in the overall aldehyde profile between *C. lectularius* and *C. hemipterus* nymphs. The percent abundance of each aldehyde was calculated based on the total amount of four aldehydes in the sample. PCA was conducted with the relative quantity data for all instars combined using the R package FactoMineR [28]. All statistical analyses were performed using R version 3.6.1 [29].

## 3. Results

### 3.1. Nymphs (Solvent Extraction of Exuviae)

All four aldehydes were consistently detected in the exuviae of all five nymphal instars of *C. lectularius*. In *C. hemipterus*, 4-oxo-(*E*)-2-octenal was not detected in two replications (20%) for the first instar, seven replications (70%) of the second instar, five replications (50%) for the third instar, and one replication (10%) for each of fourth and fifth instars. The other aldehydes were consistently detected in all samples of *C. hemipterus*. For both species, the total amount of aldehydes in the exuviae was generally lowest for the first instar and increased in each successive instar. 

Both quantity and percent abundance data from the nymphal exuviae are shown in Table 1. In *C. lectularius*, relative amounts of the four aldehydes remained similar across different instars, with (*E*)-2-octenal being most abundant (48–58%), and (*E*)-2-hexenal and 4-oxo-(*E*)-2-octenal least abundant (5–18%). The fourth aldehyde, 4-oxo-(*E*)-2-hexenal, was found in the amounts intermediate of the other three (21–33%). The percent abundance of (*E*)-2-hexenal was lowest in the first instar (5.6%) but consistently increased in successive instars, eventually reaching 17.5% in the fifth instar. Conversely, the percent abundance of 4-oxo-(*E*)-2-octenal was largest in the first instar (15.8%) but consistently decreased in successive instars, eventually declining to only 5.3% in the fifth instar. 

In *C. hemipterus*, a similar pattern was found. Relative amounts of the four aldehydes remained similar across different instars of *C. hemipterus*, with (*E*)-2-octenal being most abundant (40–65%) and (*E*)-2-hexenal and 4-oxo-(*E*)-2-octenal least abundant (0.3–24%). 4-oxo-(*E*)-2-hexenal was found in the amounts intermediate of the other three (30–38%) (Table 1). As with *C. lectularius*, the percent abundance of (*E*)-2-hexenal consistently increased across the nymphal development of *C. hemipterus* (4% in the first instar to 21% in the fifth instar). However, unlike *C. lectularius*, the amount of 4-oxo-(*E*)-2-octenal remains relatively similar across five instars, comprising 1.2% in the first instar, 0.3% in second, third, and fourth instars, and 0.7% in the fifth instar. Moreover, unlike *C. lectularius*, the relative abundance of (*E*)-2-octenal decreased over nymphal development of *C. hemipterus* (65% in the first instar to 42% in the fifth instar). 

When the quantities of an aldehyde were compared between *C. lectularius* and *C. hemipterus* within the same instar, the majority of comparisons showed significant differences (Table 2). For both first and second instars, all four aldehydes were found in greater quantities in *C. lectularius* compared to *C. hemipterus*. In all instars, *C. lectularius* had a significantly greater amount of 4-oxo-(*E*)-2-octenal than *C. hemipterus*. In all instars except the third, quantities of the two ketoaldehydes were significantly different between *C. lectularius* and *C. hemipterus*.

**Figure 1:**
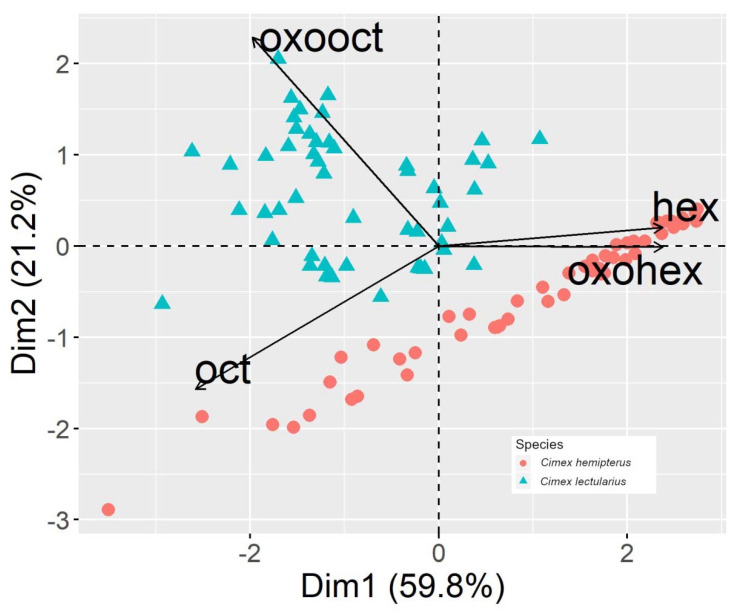
Biplot showing the first two principal components of aldehyde percent abundance in *Cimex lectularius* and *Cimex hemipterus* for all instars combined. Arrows show the impact and correlation of variables. Arrows show the contribution of each variable to the first two dimensions. Samples close to the arrows have a higher value for this variable. Variables that create acute angles are positively correlated, while those opposite each other tend to be negatively correlated. [Dim = dimension; hex = (*E*)-2-hexenal; oxohex = 4-oxo-(*E*)-2-hexenal; oct = (*E*)-2-octenal; oxooct = 4-oxo-(*E*)-2-octenal].

Principal component analyses (PCA) on the four aldehydes’ percent abundances showed clear visual separation between the two species of bed bugs. When the data from all instars were pooled together for PCA, the first two principal components (eigenvalues: PC1 = 2.39; PC2 = 0.84) accounted for 80.9% of the total variation (Figure 1). 

### 3.2. Adults

4-oxo-(*E*)-2-hexenal and 4-oxo-(*E*)-2-octenal were not detected in any of the surface extracts obtained from the adult bed bugs after brief anesthetization with carbon dioxide. Therefore, (*E*)-2-octenal and (*E*)-2-hexenal were the only two aldehydes used for calculating percent abundance. Other compounds present were not identified. In two samples (one male of each species), (*E*)-2-octenal was not detected, and thus (*E*)-2-hexenal had 100% percent abundance in those samples. The percent abundance of (*E*)-2-hexenal is reported, with (*E*)-2-octenal comprising the remaining portion.

There was a significant difference between species (*W* = 107.5, *p* = 0.0128), with adult *C. hemipterus* (57.7 ± 4.0; Mean ± SE; *n* = 20) having a greater percent abundance of (*E*)-2-hexenal than *C. lectularius* (42.1 ± 5.7; Mean ± SE; *n* = 20). There was not a significant difference (*W* = 39, *p* = 0.427) in the percent abundance of (*E*)-2-hexenal between male (52.1 ± 9.7; Mean ± SE; *n* = 10) and female (32.1 ± 4.2; Mean ± SE; *n* = 10) *C. lectularius*. Similarly, there was not a significant difference (*W* = 68, *p* = 0.186) in the percent abundance of (*E*)-2-hexenal between male (55.7 ± 6.8; Mean ± SE; *n* = 10) and female (59.7 ± 4.0; Mean ± SE; *n* = 10) *C. hemipterus*. 

## 4. Discussion

In all nymphal stages (*E*)-2-octenal was found in the highest amounts. Among the four aldehydes analyzed, 4-oxo-(*E*)-2-octenal was dramatically and consistently different between *C. lectularius* and *C. hemipterus*, consistent with findings by Liedtke et al. [22]. We found that the amount of the four aldehydes in the two earliest instars were all significantly different between the two species. Except for third instars, the amounts of the two ketoaldehydes were always significantly different between species, consistent with results reported by Liedtke et al. [22]. Though Liedtke et al. [22] found that the amounts of (*E*)-2-hexenal and (*E*)-2-octenal emitted by nymphs did not differ between species, we found significant differences between species in first, second and fourth instars, but not in the third or fifth instars. As Liedtke et al. [22] collected aldehydes from the headspace of only late-stage instars, this likely explains why they did not see such differences. 

The total quantity of aldehydes increased from first to fifth instars of *C. lectularius*, and *C. hemipterus* by approximately 22 and 59 times, respectively. The average total quantities of the four aldehydes found in *C. lectularius* fifth instar exuviae (6.73 μg) were higher than those reported by Choe et al. [9] (2.11 μg). Though similar methods were used to quantify aldehydes, the discrepancy may be explained by different time intervals between the shedding of exuviae and the chemical extraction. For example, the current study extracted freshly shed exuviae, while the exuviae used by Choe et al. [9] were aged at least for seven days prior to the extraction. If exuviae lose a large fraction of their aldehyde contents shortly after molting, the freshly shed exuviae might serve as a source of the aldehyde pheromone with significant behavioral implications, such as the forming aggregations at harborage sites. All four aldehydes might volatilize/dissipate in similar rates. Choe et al. [9] estimated that these aldehydes in the fifth instar exuviae (*C. lectularius*) reduced by approximately 1.4–2.3% per day. This possibility is further supported by a similar pattern in the ratio of the four aldehydes we found in fifth instar *C. lectularius* exuviae compared with those reported by Choe et al. [9]. 

Due to the quantification of only one strain for each species, some caution is warranted when interpreting our findings. However, based on other studies investigating the aldehydes in other strains of these two species, similar aldehyde ratios are apparent. For example, comparing the ratio of the most and least prevalent of the four aldehydes [(*E*)-2-octenal and 4-oxo-(*E*)-2-octenal], we find similarities. Our findings of a ratio of 11.6:1 for fifth instar *C. lectularius* are similar to the 12.6:1 ratio previously reported by Feldlaufer et al. [14]. Choe et al. [9] and Liedtke et al. [22] reported ratios of 6.6:1 and 6.2:1, respectively. These discrepancies may be a result of the different methodologies used or differences between bed bug strains. However, these ratios all follow the same pattern of (*E*)-2-octenal being several times the amount of 4-oxo-(*E*)-2-octenal. Our study reports a 60:1 ratio between (*E*)-2-octenal and 4-oxo-(*E*)-2-octenal in *C. hemipterus*, which follows a similar pattern with the 101:1 ratio previously reported by Liedtke et al. [22].

Based on the percent abundance of 4-oxo-(*E*)-2-octenal in exuviae, two bed bug species can be readily identified. The differences in both quantity and ratio of these four aldehydes in the exuviae of *C. lectularius* and *C. hemipterus* may provide a method of distinguishing these species based on chemical differences. This approach may be useful when insect specimens are not present or when morphological information is missing due to damaged specimens. The chemical identification approach based on aldehydes can be supplemented by additional information based on cuticular hydrocarbons (CHCs) if the CHC profile of *C. hemipterus* is substantially different from that of *C. lectularius* as reported by Feldlaufer and Blomquist [30]. 

(*E*)-2-octenal and (*E*)-2-hexenal were the primary aldehydes in adult bed bugs extracts for both species. The ketoaldehydes 4-oxo-(*E*)-2-hexenal and 4-oxo-(*E*)-2-octenal were not detected in the surface extracts of adult bed bugs. This finding corroborates previous work reporting that nymphs produce these ketoaldehydes in the dorsal abdominal glands for both species [14,22]. As adult bed bugs lack the dorsal abdominal glands [1,16], this explains the absence of these ketoaldehydes in adult bed bugs. Based on the relative abundance data of (*E*)-2-octenal and (*E*)-2-hexenal, we found significant differences between adults of *C. lectularius* and *C. hemipterus*. *Cimex hemipterus* had a greater proportion of (*E*)-2-hexenal than *C. lectularius*, and the proportion of (*E*)-2-octenal was greater in *C. lectularius* compared to *C. hemipterus*. However, Liedtke et al. [22] did not find significant differences between *C. lectularius* and *C. hemipterus* in the ratio of (*E*)-2-hexenal and (*E*)-2-octenal emitted by the adults based on the sampling of the headspace volatiles. Liedtke et al. [22] found that the ratio of (*E*)-2-hexenal:(*E*)-2-octenal in *C. hemipterus* adult females to be 59∶41 and 45∶54 in adult males. In *C. lectularius* adults, Liedtke et al. [22] found the same ratio to be 52∶48 for females and 68∶32 for males. We found this ratio for *C. hemipterus* adults to be 60:40 in females and 56:44 in males, while in *C. lectularius*, the ratio was 32:68 in females and 52:48 in males. In comparing these studies, we see the ratios reported for *C. hemipterus* adult females were nearly identical between the studies, while ratios for others differ significantly. This discrepancy may be due to differences in bed bug strains or the different sampling methods used (surface extraction vs. headspace collection).

This work provides the first systematic quantification of four aldehydes [(*E*)-2-hexenal, 4-oxo-(*E*)-2-hexenal, (*E*)-2-octenal and 4-oxo-(*E*)-2-octenal] for each of the five nymphal stages for both *C. lectularius* and *C. hemipterus*. Additionally, the percent abundance of (*E*)-2-hexenal and (*E*)-2-octenal was determined for adults of each species. Our findings showed that even though both species produced all of the aldehydes investigated in the current study, aldehyde blends of *C. lectularius* and *C. hemipterus* were quantitatively distinctive. The behavioral study of these synanthropic bed bug species towards these aldehyde mixtures (heterospecific or conspecific blends) might help us further understand the potential significance of the distinctive blend ratio in the intraspecific and/or interspecific communication.

## 5. Conclusions

The amounts of four aldehydes [(*E*)-2-hexenal, 4-oxo-(*E*)-2-hexenal, (*E*)-2-octenal, and 4-oxo-(*E*)-2-octenal] differ significantly between *C. lectularius* and *C. hemipterus* in the earliest two instars. The amounts of the two ketoaldehydes vary among the two species, except for third instar. Adult *C. hemipterus* have a higher percent abundance of (*E*)-2-hexenal than *C. lectularius*. We found no differences in adults’ aldehyde ratio based on sex for both *C. lectularius* and *C. hemipterus*.

## Figures and Tables

**Table 1:** Mean quantity (µg/exuvia; *n* = 10) and percent abundance (mean ± SE) of four aldehydes detected in freshly shed exuviae of first through fifth instar *Cimex lectularius* and *Cimex hemipterus* nymphs. Different letters across rows indicate significance differences in each compound across the five instars (*p* < 0.05; Dunn’s Multiple Comparisons) (SE = Standard error).

***Cimex lectularius***										
**Compound**	**Instar**									
	**First**		**Second**		**Third**		**Fourth**		**Fifth**	
	**Mean ± SE**	**% abundance**	**Mean ± SE**	**% abundance**	**Mean ± SE**	**% abundance**	**Mean ± SE**	**% abundance**	**Mean ± SE**	**% abundance**
(*E*)-2-hexenal	0.018 ± 0.002 a	5.6 ± 0.3	0.074 ± 0.007 ab	7.6 ± 0.8	0.158 ± 0.012 bc	8.7 ± 0.8	0.315 ± 0.049 c	11.6 ± 1.2	1.326 ± 0.292 c	17.5 ± 1.8
4-oxo-(*E*)-2-hexenal	0.083 ± 0.005 a	26.9 ± 0.4	0.219 ± 0.020 a	21.5 ± 1.3	0.628 ± 0.040 b	33.2 ± 1.3	0.690 ± 0.101 b	24.7 ± 1.7	1.472 ± 0.293 b	21.1 ± 0.9
(*E*)-2-octenal	0.159 ± 0.010 a	51.7 ± 0.4	0.586 ± 0.043 ab	57.6 ± 0.9	0.951 ± 0.103 bc	48.2 ± 1.4	1.496 ± 0.154 cd	55.9 ± 1.6	3.624 ± 0.646 d	56.2 ± 1.7
4-oxo-(*E*)-2-octenal	0.048 ± 0.003 a	15.8 ± 0.5	0.137 ± 0.013 b	13.3 ± 0.6	0.198 ± 0.026 bc	9.9 ± 0.6	0.217 ± 0.031 bc	7.8 ± 0.5	0.311 ± 0.053 c	5.3 ± 0.6
***Cimex hemipterus***										
**Compound**	**Instar**									
	**First**		**Second**		**Third**		**Fourth**		**Fifth**	
	**Mean ± SE**	**% abundance**	**Mean ± SE**	**% abundance**	**Mean ± SE**	**% abundance**	**Mean ± SE**	**% abundance**	**Mean ± SE**	**% abundance**
(*E*)-2-hexenal	0.006 ± 0.002 a	4.0 ± 0.6	0.022 ± 0.007 ab	7.7 ± 1.2	0.255 ± 0.054 bc	15.7 ± 1.2	0.854 ± 0.148 cd	24.3 ± 1.4	1.460 ± 0.174 d	20.6 ± 1.4
4-oxo-(*E*)-2-hexenal	0.038 ± 0.008 a	30.0 ± 1.4	0.079 ± 0.019 a	32.6 ± 2.7	0.565 ± 0.083 b	37.9 ± 1.1	1.178 ± 0.133 bc	35.6 ± 1.1	2.639 ± 0.269 c	37.1 ± 1.1
(*E*)-2-octenal	0.075 ± 0.013 a	64.8 ± 1.8	0.119 ± 0.022 a	59.4 ± 3.4	0.658 ± 0.075 b	46.2 ± 1.6	1.297 ± 0.135 bc	39.8 ± 1.0	2.922 ± 0.285 c	41.6 ± 0.9
4-oxo-(*E*)-2-octenal	0.001 ± 0.0003 ab	1.2 ± 0.3	0.001 ± 0.0005 b	0.3 ± 0.2	0.004 ± 0.002 ab	0.3 ± 0.1	0.010 ± 0.002 bc	0.3 ± 0.1	0.049 ± 0.009 c	0.7 ± 0.1

**Table 2:** Quantity (µg/exuvia; *n* = 10) comparisons between nymphal *Cimex lectularius* and *Cimex hemipterus* for each aldehyde compound within an instar. Significant differences (Wilcoxon rank-sum test) between species are indicated in the *C. hemipterus* column (* *p* < 0.05, ** *p* < 0.01, *** *p* < 0.001).

Instar	Compound	*Cimex lectularius* (Mean ± SE; µg/exuvia)	*Cimex hemipterus* (Mean ± SE; µg/exuvia)
First	(*E*)-2-hexenal	0.018 ± 0.002	0.006 ± 0.002 ***
	4-oxo-(*E*)-2-hexenal	0.083 ± 0.005	0.038 ± 0.008 **
	(*E*)-2-octenal	0.159 ± 0.010	0.075 ± 0.013 ***
	4-oxo-(*E*)-2-octenal	0.048 ± 0.003	0.001 ± 0.0003 ***
Second	(*E*)-2-hexenal	0.074 ± 0.007	0.022 ± 0.007 **
	4-oxo-(*E*)-2-hexenal	0.219 ± 0.020	0.079 ± 0.019 ***
	(*E*)-2-octenal	0.586 ± 0.043	0.119 ± 0.022 ***
	4-oxo-(*E*)-2-octenal	0.137 ± 0.013	0.001 ± 0.0005 ***
Third	(*E*)-2-hexenal	0.158 ± 0.012	0.255 ± 0.054
	4-oxo-(*E*)-2-hexenal	0.628 ± 0.040	0.565 ± 0.083
	(*E*)-2-octenal	0.951 ± 0.103	0.658 ± 0.075
	4-oxo-(*E*)-2-octenal	0.198 ± 0.026	0.004 ± 0.002 ***
Fourth	(*E*)-2-hexenal	0.315 ± 0.049	0.854 ± 0.148 **
	4-oxo-(*E*)-2-hexenal	0.690 ± 0.101	1.178 ± 0.133 *
	(*E*)-2-octenal	1.496 ± 0.154	1.297 ± 0.135
	4-oxo-(*E*)-2-octenal	0.217 ± 0.031	0.010 ± 0.002 ***
Fifth	(*E*)-2-hexenal	1.326 ± 0.292	1.460 ± 0.174
	4-oxo-(*E*)-2-hexenal	1.472 ± 0.293	2.639 ± 0.269 *
	(*E*)-2-octenal	3.624 ± 0.646	2.922 ± 0.285
	4-oxo-(*E*)-2-octenal	0.311 ± 0.053	0.049 ± 0.009 ***
